# A Novel and Effective Model to Predict Skip Metastasis in Papillary Thyroid Carcinoma Based on a Support Vector Machine

**DOI:** 10.3389/fendo.2022.916121

**Published:** 2022-07-05

**Authors:** Shuting Zhu, Qingxuan Wang, Danni Zheng, Lei Zhu, Zheng Zhou, Shiying Xu, Binbin Shi, Cong Jin, Guowan Zheng, Yefeng Cai

**Affiliations:** ^1^ Department of Ultrasound, The First Medical Center of Chinese People’s Liberation Army (PLA) General Hospital, Beijing, China; ^2^ Department of Thyroid Surgery, The First Affiliated Hospital of Wenzhou Medical University, Wenzhou, China; ^3^ Department of Breast Surgery, The First Affiliated Hospital of Wenzhou Medical University, Wenzhou, China; ^4^ Thyroid Surgery Department, The Fifth Hospital Affiliated to Wenzhou Medical University, Lishui Central Hospital, Lishui, China; ^5^ Department of Head and Neck Surgery, Bengbu Medical College Graduate School, Anhui, China; ^6^ Zhejiang Chinese Medical University, The Second Clinical Medical, Hangzhou, China; ^7^ Department of Medical Ultrasound, the First Affiliated Hospital of Wenzhou Medical University, Wenzhou, China; ^8^ Department of Head and Neck Surgery, Otolaryngology & Head and Neck Center, Cancer Center, Zhejiang Provincial People’s Hospital (Affiliated People’s Hospital, Hangzhou Medical College), Hangzhou, China; ^9^ Key Laboratory of Endocrine Gland Diseases of Zhejiang Province, Hangzhou, China

**Keywords:** predict model, skip lymph node metastasis, papillary thyroid carcinoma, support vector, BRAF mutation

## Abstract

**Introduction:**

Skip metastasis, referred to as lymph node metastases to the lateral neck compartment without involvement of the central compartment, is generally unpredictable in papillary thyroid carcinoma (PTC). This study aims to establish an effective predictive model for skip metastasis in PTC.

**Meterials and Methods:**

Retrospective analysis was performed of clinical samples from 18192 patients diagnosed with thyroid cancer between 2016 to 2020. The First Affiliated Hospital of Wenzhou Medical University. The lateral lymph node metastasis was occureed in the training set (630 PTC patients) and validation set (189 PTC patients). The univariate and multivariate analyses were performed to detect the predictors of skip metastasis and the support vector machine (SVM) was used to establish a model to predict skip metastasis.

**Results:**

The rate of skip metastasis was 13.3% (84/631). Tumor size (≤10 mm), upper location, Hashimoto’s thyroiditis, extrathyroidal extension, absence of BRAFV600E mutation, and less number of central lymph node dissection were considered as independent predictors of skip metastasis in PTC. For the training set, these predictors performed with 91.7% accuracy, 86.4% sensitivity, 92.2% specificity, 45.2% positive predictive value (PPV), and 98.9% negative predictive value (NPV) in the model. Meanwhile, these predictors showed 91.5% accuracy,71.4% sensitivity, 93.1% specificity, 45.5% PPV, and 97.6% NPV in validation set.

**Conclusion:**

This study screened the predictors of the skip lateral lymph node metastasis and to establish an effective and economic predictive model for skip metastasis in PTC. The model can accurately distinguish the skip metastasis in PTC using a simple and affordable method, which may have potential for daily clinical application in the future.

## Introduction

The incidence of thyroid cancer has remarkably increased in the last 40 years worldwide primarily because of the increase in the incidence of papillary thyroid carcinoma (PTC) (1). Papillary thyroid carcinoma, a common endocrine malignancy, accounts for 90% of thyroid cancer (2, 3). The dissemination of PTC cells follows a predictable stepwise pattern, metastasizing from the central lymph node (CLN) compartment to the lateral lymph node (LLN) compartment (4, 5). Thus, CLN is considered as the first step of lymphatic drainage of PTC in most previous studies (6, 7). However, lateral lymph node metastasis (LLNM) of PTC without central lymph node metastasis (CLNM) is also found (8). This unpredictable pattern of LNM is known as skip metastasis of PTC.

Skip metastasis is not unique to PTC. This metastatic form without stepwise spread also exists in gastric, colorectal, lung, and breast carcinoma (9–12). Giving inadequate attention to any tumor may adversely affect treatment outcomes and cause poor prognosis. In addition, skip metastasis is not a rare phenomenon. For example, skip metastasis of gastrointestinal cancer is frequent because the location of the lymphatic drainage tract is complicated (13). Skip metastasis is also common in PTC, and its incidence ranges from 5% to 25% (the data are obtained from different samples) (14).

Some studies have suggested that regional lymph node metastasis (LNM) is a vital factor in poor prognosis of patients with PTC (15). In addition, LLNM has shown stronger connection with locoregional recurrence and unfavorable prognosis than CLNM in patients with PTC (16). However, whether skip LNM is associated with a relatively poor prognosis remains unclear (14). Some studies show that LNM can increase the risk of lymph node recurrence, and the reoperation of recurrence can increase operative complications and medical costs to some degree (17–19). Therefore, skip metastasis in clinical diagnosis and treatment must be given importance to avoid risks as much as possible.

Preoperative ultrasonographic evaluation and biopsy results of cervical lymph nodes primarily determined the operative mode of PTC. Suspicious and/or biopsy-proven nodal metastases should be treated with a formal compartmental resection (20). Therefore, the neglect of skip metastasis may lead to incomplete surgical resection. However, few effective methods to predict skip metastasis in PTC were identified. Therefore, our study investigated the frequency and risk factors for skip metastasis and established a model to predict skip metastasis, make a reasonable surgical project or follow-up plan, and achieve treatment effectiveness.

## Materials and Methods

### Patients

**Figure 1:**
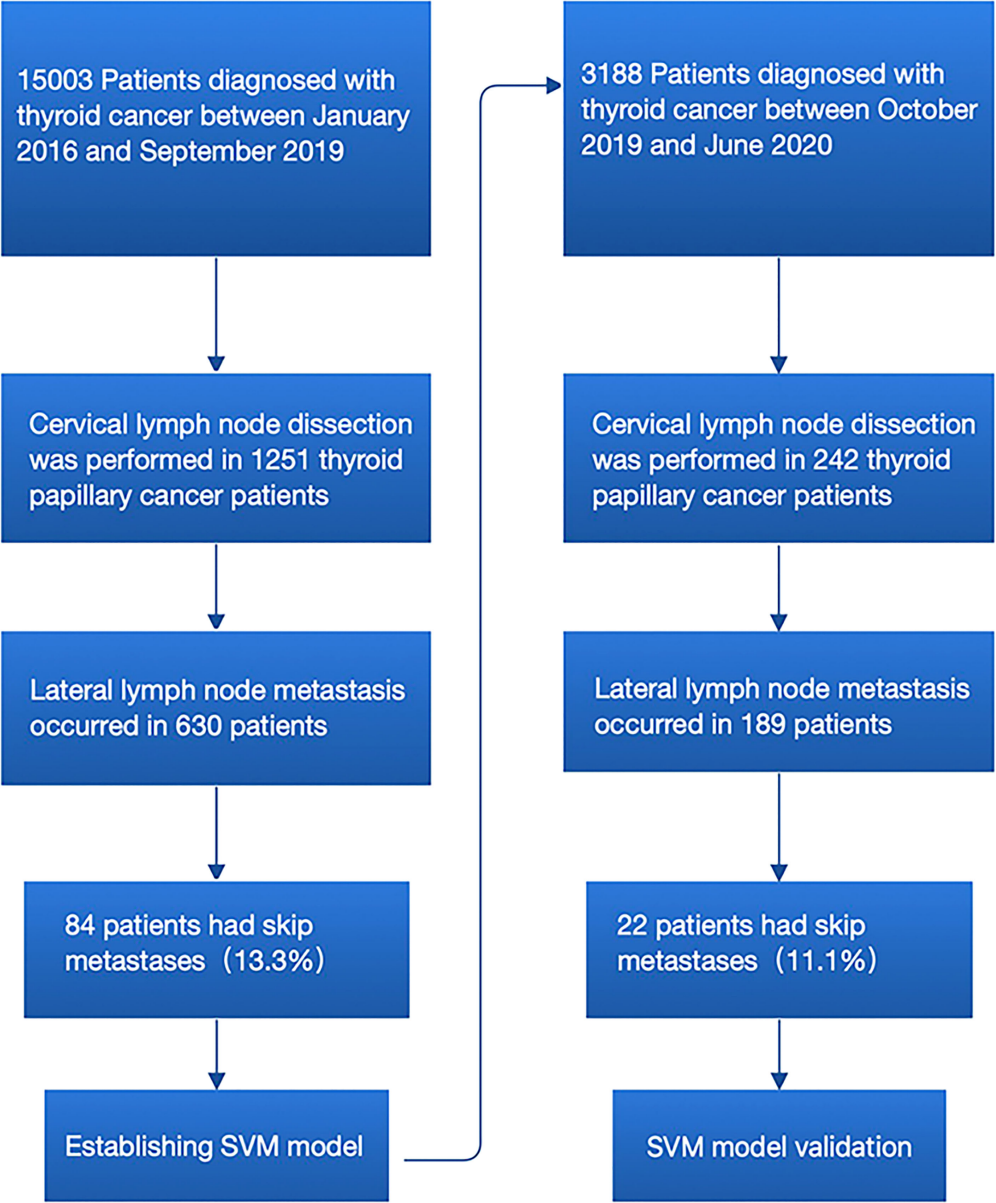
Flowchart for the process of establishment and validation of skip metastasis prediction model of PTC.

Two sets of patients were included in the training set and validation set. Patients in the training set were used to investigate the predictors of skip metastasis and to establish predictive models, whereas patients in the validation set were used to examine the efficacy of the predictive models. A total 631 patients with PTC who received initial thyroidectomy with central lymph node dissection (CLND) plus lateral lymph node dissection (LLND) at the First Affiliated Hospital of Wenzhou Medical University during January 2016 to September 2019 were included in the training set. Meanwhile, 189 external patients who received the same surgical procedure during October 2019 to June 2020 were included in the validation set (**Figure 1**). All of the patients were identified as PTC with LLNM by postoperative pathological report. Each set was further divided into the skip metastasis group and non-skip metastasis group.

The exclusion criteria were as follows: insufficient data or unknown clinicopathologic profile, undetermined histology, history of neck surgery or irradiation, negative lateral lymph node metastases, and other types of thyroid cancer (follicular thyroid cancer, medullary thyroid cancer, anaplastic thyroid cancer, etc.). The study obtained approval from the Ethics Committee of the First Affiliated Hospital of Wenzhou Medical University.

### Operational Approach

The operation was performed by the same experienced surgical team and the same standard procedure. The scope of CLND was up to the thyroid cartilage notch, down to the carotid sheath, posterior to the prevertebral fascia, and final to the innominate vein. In this study, the lateral lymph node compartment included levels II, III, IV, and V. The area was drawn up to the posterior belly of the digastric muscle, down to the subclavian vein, and next to the anterior border of the trapezius muscle.

### Parameter Analysis

Our study collected information through electronic clinical records, pathological records, and preoperative ultrasonographic images. Patient’s baseline information (gender and age) and clinical data (hashimoto’s thyroiditis, body mass index [BMI], metabolic syndrome, hypertension, and diabetes) were analyzed. In addition, the thyroid nodule sonographic features were evaluated and analyzed, by three experienced sonographers, on the basis of the size of tumor, location, margin, taller-than-wide shape, calcification, vascularization, multifocality, extrathyroidal extension, and the information about cervical lymph node. The information about BRAF^V600E^ mutation was collected as well.

We analyzed the largest tumor or the most suspicious dominant nodule of the thyroid, when multiple nodules were found. Standard preoperative sonography of PTC tumor boundaries was performed to determine the size, and taller-than-wide shape. Moreover, the thyroid gland was trisected vertically, dividing it into three locations (upper, middle, and lower) to determine the location of tumors. Calcification was defined as the hyperechoic spots with or without acoustic shadows or as simple fine acoustic shadows in ultrasound (21). The multifocality of a tumor was confirmed when more than one mass was found based on the preoperative ultrasound examination or the intraoperative pathological diagnose (22). Extrathyroidal extension was defined as the tumor perimeter in contact with >25% of the thyroid capsule in a malignant lesion or the loss of the capsule line (23). The diagnosis of hashimoto’s thyroiditis relies on the demonstration of circulating antibodies to thyroid antigens (mainly anti-thyroglobulin >30U/ml (normal range is 0-4U/ml); anti-thyroperoxidase (anti-TPO) >30U/ml) (normal range is 0-9U/ml) and reduced echogenicity and prominent heterogeneous thyroid parenchyma on thyroid sonogram in a patient with proper clinical features (24). The information of cervical lymph node was obtained from the final pathological reports.

### Model Development

A support vector machine (SVM) was used for establishing skip metastasis prediction models. The prediction models were established by Libsvm 3.20 with the modeling platform of MATLAB 2019a. The C-SVC, RBF kernel functions and grid search method were used to debug the model. The grid c bound, grid c step, grid g bound, and grid g step were −8 to 8, 0.5, −8 to 8, and 0.5, respectively. The fold number of cross-validation was 5. As we divided patients into two groups, the positive value indicated skip metastasis, and the negative value indicated non-skip metastasis.


$$P\left(label\right)=sgn(\sum_{n=1}^{\infty }ni=(0wi\;exp(-ganma\left|xi-x\right|2+b)))$$

### Statistics

The data showed a normal distribution through an independent two-sample Student’s t-test. Statistical analysis was performed using SPSS version 26.0 (SPSS, Chicago, IL, USA). Categorical variables were compared with the results of Chi-square test or Fisher’s exact test. The difference between two sides was statistically significant, when *P* value ≤ 0.05. All factors with significant associations based on univariate analysis were included for multivariate analysis using forward stepwise selection.

## Results

### The Baseline Information in the Training and Validation Set

1. Gender: The female percent is 67.6% (426/630) in the training set and 61.9% (117/189) in the validation set.
2. Age: The average age of training set is 45.28 ± 12.48 years (range, 17–84 years), and that of the validation set is 46.69 ± 12.11 years (range, 21–74 years).
3. The size of a primary tumor: the mean size is 14.25 ± 8.88 mm (range, 1–67mm) and 12.25 ± 7.74 mm (range, 1–40mm) in the training set and validation set, respectively.
4. The mean number of total harvested CLN is 7.57 ± 5.71 (range, 1–28) in the training set and 6.95 ± 5.18 (range, 1–35) in the validation set.
5. The rate of skip metastasis is 13.3% (84/630) in the training test and 11.6% (22/189) in the validation set.
6. The rate of BRAF^V600E^ mutation is 75.1% (473/630) in the training test and 82.0% (155/189) in the validation set (**Table S1**).

### Predictors of Skip Metastasis of PTC in the Training Set

Data from the training set were used to investigate the predictors of skip metastasis in PTC. The first step of analysis was comparing the clinicopathological factors between the skip metastasis group and non-skip metastasis by univariate analysis. The following characteristics of patients were more likely observed in the skip metastasis group (**Table 1**): age>45 (*P*=0.008), tumor size ≤ 10 mm (*P*=0.001), Hashimoto’s thyroiditis (*P*=0.009), upper tumor location (*P*<0.001), well-defined margin (*P*=0.05), extrathyroidal extension (*P*=0.002), absence of BRAF^V600E^ mutations (*P*=0.006), BMI≥25 (*P*=0.024), and less number of CLND (the mean number of the skip metastasis was 5.17 ± 3.81, *P*=0.001).

**Table 1:** Comparison of clinicopathological factors between skip metastasis and non-skip metastasis with PTC. .

Characteristics	Skip metastasis		F	P value
	Present (n=84)	Absent (n=546)		
Sex			2.412	0.12
Male (n, %)	21 (25.0%)	183 (33.5%)		
Female (n, %)	63 (75.0%)	363 (66.5%)		
Age at diagnosis (years)	49.55 ± 11.73	44.62 ± 12.48	6.995	0.008^*^
≤45 (n, %)	33 (39.3%)	299 (54.8%)		
>45 (n, %)	51 (60.7%)	247 (45.2%)		
Tumor size (mm)	12.83 ± 8.85	14.47 ± 8.87	10.859	0.001^*^
≤10 (n, %)	50 (59.5%)	217 (39.9%)		
>10 (n, %)	34 (40.5%)	329 (60.1%)		
Multifocality (n,%)			3.249	0.071
Yes (n, %)	21 (25%)	191 (35.0%)		
No (n, %)	63 (75%)	355 (65.0%)		
Hashimoto’s thyroiditis			6.784	0.009^*^
Yes (n, %)	29 (34.5%)	118 (21.6%)		
No (n, %)	55 (65.5%)	428 (78.4%)		
Tumor location			15.066	<0.001^*^
Upper (n, %)	41 (48.8%)	152 (27.8%)		
Lower/middle/isthmus (n, %)	43 (51.2%)	394 (72.2%)		
Taller than wide			0.74	0.39
Yes (n, %)	17 (20.2%)	134 (24.5%)		
No (n, %)	67 (79.7%)	412 (73.4%)		
Calcification			0.873	0.35
Calcification (n, %)	68 (81.0%)	462 (84.6%)		
Absence (n, %)	16 (19.0%)	84 (15.4%)		
Margin			3.369	0.05^*^
Well-defined (n, %)	63 (73.8%)	333 (61.0%)		
Without well-defined margin (n, %)	21 (26.2%)	213 (38.5%)		
Shape			1.365	0.243
Regular (n, %)	20 (23.8%)	164 (30.0%)		
Irregular (n, %)	64 (76.2%)	382 (70.0%)		
Extrathyroidal extension			10.077	0.002^*^
Yes (n, %)	35 (41.7%)	137 (25.1%)		
No (n, %)	49 (58.3%)	409 (74.9%)		
Vascularization			1.715	0.19
Present (n, %)	13 (15.5%)	58 (10.6%)		
Absence (n, %)	71 (84.5%)	488 (89.4%)		
BRAF^V600E^			7.44	0.006^*^
Mutation (n, %)	53 (63.1%)	420 (76.9%)		
No mutation (n, %)	31 (26.7%)	126 (23.1%)		
BMI			5.065	0.024^*^
<25 (n, %)	50 (59.5%)	391 (71.6%)		
≥25 (n, %)	34 (40.5%)	155 (28.4%)		
hypertension			0.963	0.326
Yes (n, %)	19 (22.6%)	99 (18.3%)		
No (n, %)	65 (77.3%)	447 (81.9%)		
diabetes			0.856	0.355
Yes (n, %)	6 (7.1%)	26 (4.8%)		
No (n, %)	78 (92.9%)	520 (95.2%)		
Metabolic syndrome			2.097	0.148
Yes (n, %)	18 (21.4%)	83 (15.2%)		
No (n, %)	66 (78.6%)	463 (84.8%)		
Cervical lymphadenopathy			0.826	0.363
Yes (n, %)	54 (64.3%)	378 (69.2%)		
No (n, %)	30 (35.7%)	168 (30.8%)		
Central lymph nodes dissected number	5.17 ± 3.81	6.73 ± 3.79	3.269	0.001^*^

^*^P value ≤ 0.05.

Further multivariate analysis showed that tumor size (≤10 mm; OR 2.331; *P*=0.003; 95% CI 1.342–4.049), upper location (OR 2.595; *P*<0.001; CI 1.535–4.388), Hashimoto’s thyroiditis (OR 3.968; *P*<0.001; CI 2.178–7.229), extrathyroidal extension (OR 3.506; *P*<0.001; CI 1.962–6.265), absence of BRAF^V600E^ mutation (OR 0.373; *P*<0.001; CI 0.212–0.658), and less number of CLND (OR 1.304; *P*<0.001; CI 1.193–1.423) were independent predictors of skip metastasis in PTC (**Table 2**). As mentioned previously, a difference in BMI values and well-defined margin was found between the skip metastasis group and non-skip metastasis group in univariate analysis. No further evidence indicated that such characteristics were independent predictors of skip metastasis in PTC.

**Table 2:** Multivariate analysis between the clinicopathologic factors and skip metastasis.

Characteristic	OR	95% CI	P value
Tumor size (≤10 mm)	2.331	1.342-4.049	0.003^*^
Extrathyroidal extension	3.506	1.962-6.265	<0.001^*^
Hashimoto’s thyroiditis	3.968	2.178-7.229	<0.001^*^
BRAFV600E mutation	0.373	0.212-0.658	0.001^*^
locating in the upper pole	2.595	1.535-4.388	<0.001^*^
Less CLND number	1.304	1.193-1.423	<0.001^*^

^*^P value ≤ 0.05.

### Information about LLN Between the Skip Metastasis Group and Non-skip Metastasis Group

In addition, the information about LLN between the skip metastasis group and non-skip metastasis group was compared (**Table 3**). We found that the number of LLNM of skip metastasis was less than that of non-skip metastasis (mean 2.04 ± 1.71 and 3.30 ± 2.59, respectively; *P*<0.001). No contralateral LLNM was found in 84 patients with skip metastasis, whereas 26 patients in the non-skip metastasis group showed contralateral LLNM. Therefore, the difference in contralateral LLNM was found between the two groups (*P*=0.037). By contrast, no difference was found in the number of LLND between the two groups (*P*=0.509).

**Table 3:** Comparison of LLN between skip metastasis and non-skip metastasis with PTC.

	Skip metastasis		F/t value	P Value
	Present (n = 84)	Absent (n = 546)		
The number of LLND (Mean ± SD)	10.79 ± 7.34	11.43 ± 8.49	-0.611	0.509
The number of LLNM (Mean ± SD)	2.04 ± 1.71	3.30 ± 2.59	-5.816	<0.001^*^
Contralateral LLNM			Fish Test	0.037^*^
Yes	0	26		
No	84	526		

^*^P value ≤ 0.05.

### Training Set: Establishing a Model to Predict Skip Metastasis

**Figure 2:**
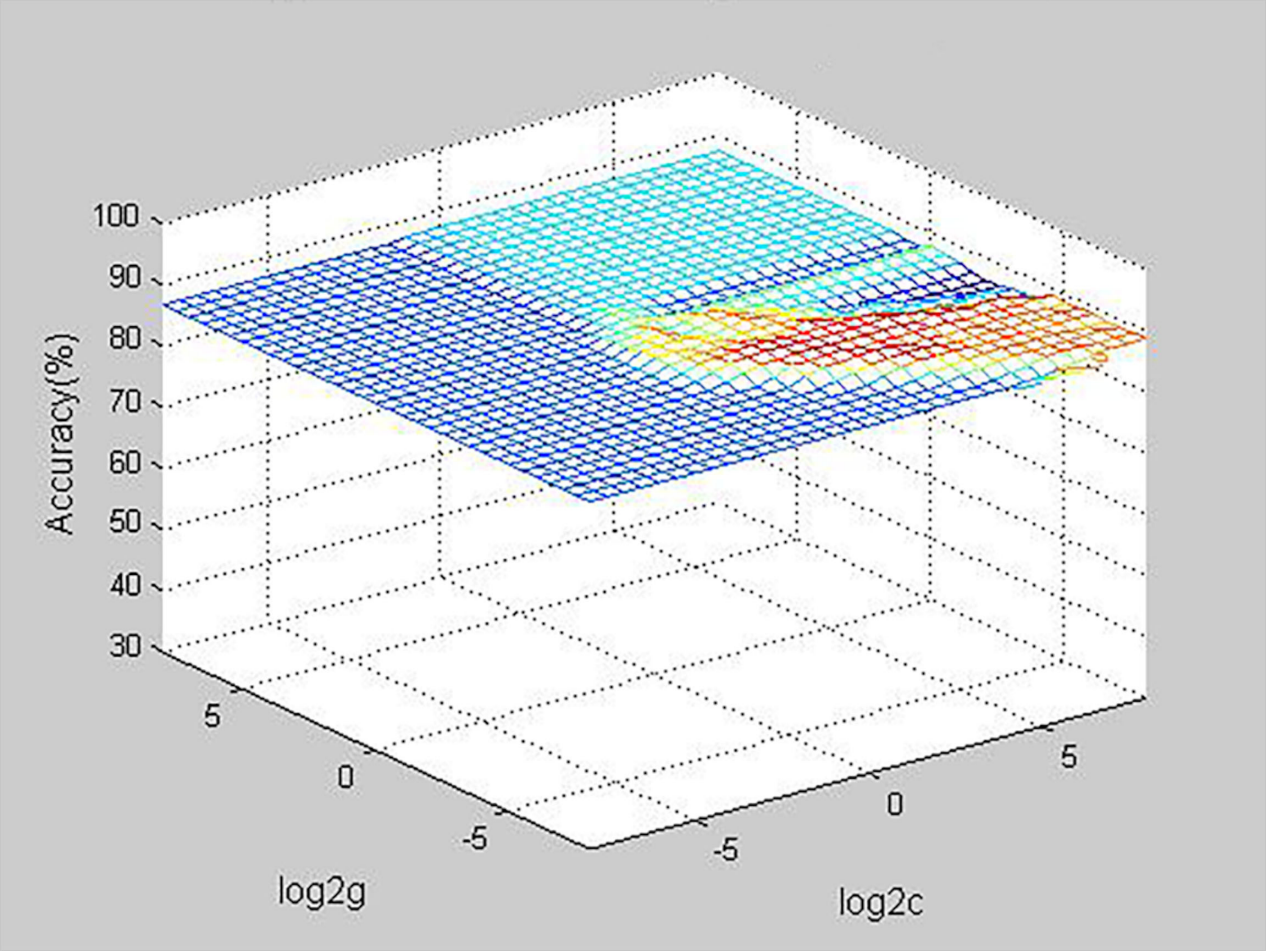
D view of the predictive value for the model (the best parameter c is 16, the parameter g is 0.0884, the parameter b is -0.5531).

The predictive panel was built using SVM, which combined the six independent predictors (tumor size, location, Hashimoto’s thyroiditis, extrathyroidal extension, BRAF^V600E^ mutation, and number of CLND). In addition, skip metastasis in the training set was predicted by this panel (**Table 4**). These predictors performed with high 91.7% accuracy in the panel (sensibility: 86.4%, specificity: 92.2%, positive predictive value [PPV]: 45.2%, negative predictive value [NPV]: 98.9%), albeit the accuracy of each individual factor was low (**Table 5**). Parameters c, g, and b were 16, 0.0884, and −0.5531, respectively, which were used to make the 3D view (**Figure 2**).

**Table 4:** The predictive model and pathological diagnosis for skip metastasis in both training and validation set.

Predicting Model				
		Positive	Negative	Total
Pathological diagnosis in Train set	positive	38	46	84
	negative	6	540	546
	total	44	586	630
Pathological diagnosis in Test set	positive	10	12	22
	negative	4	163	167
	total	14	175	189

**Table 5:** The predictive value for the model and independent predictors.

Independent predictors	sensitivity	Specificity	PPV^*^	NPV^*^	accuracy
Size	13.8%	87.0%	41.7%	60.1%	57.6%
Location	21.2%	90.2%	48.8%	72.2%	69.0%
Number of central lymph node	20.1%	94.3%	79.8%	51.3%	55.1%
Extrathyroidal extension	20.3%	89.3%	41.7%	74.9%	70.5%
BRAF^V600E^ mutation	19.7%	88.8%	36.9%	76.9%	71.6%
Hashimoto’s thyroiditis	19.7%	88.6%	34.5%	78.4%	72.5%
The model	86.4%	92.2%	45.2%	98.9%	91.7%

^*^PPV, positive predictive value; NPV, negative predictive value.

### Validation Set: Validating the Model in External Independent Samples

**Figure 3:**
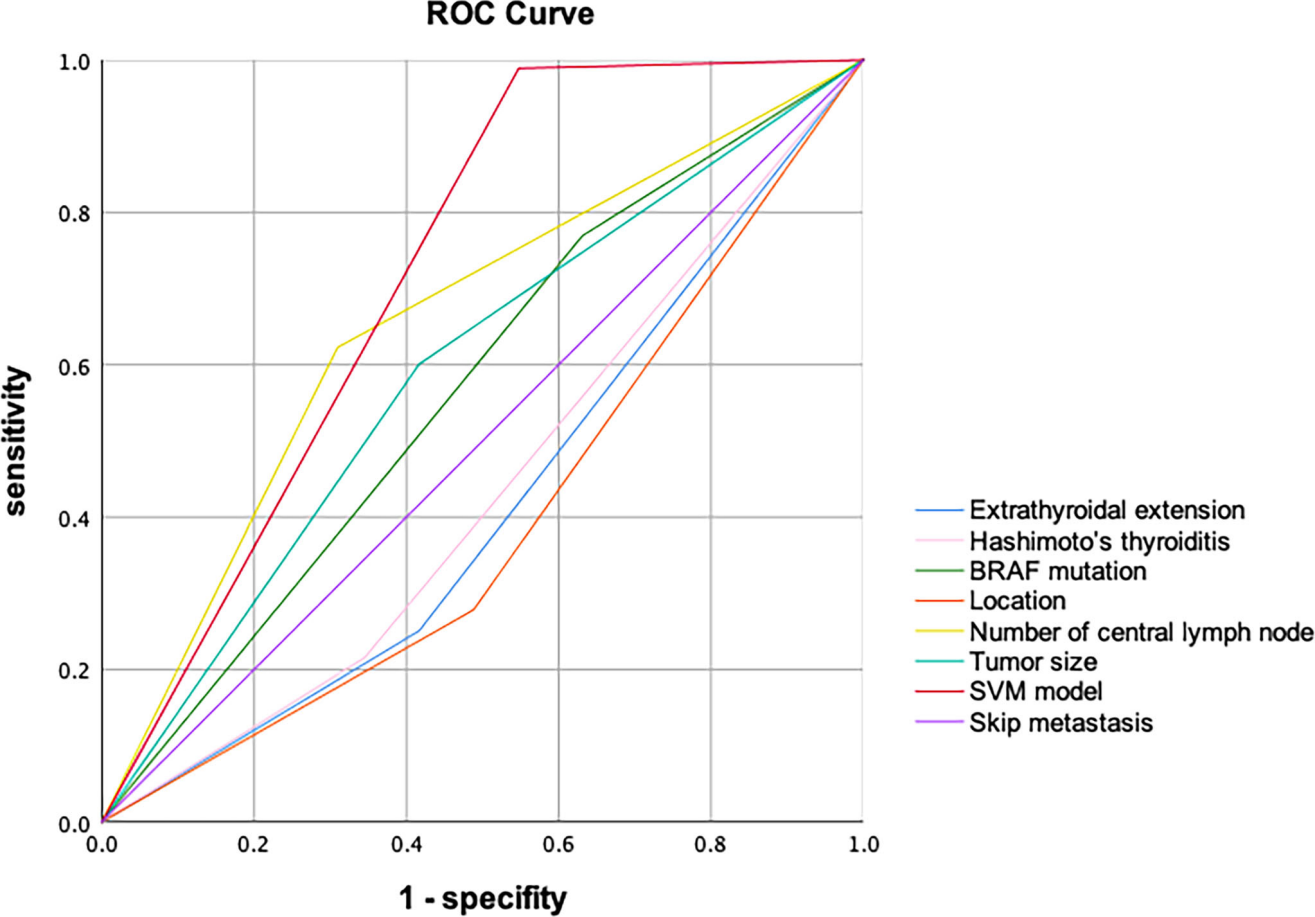
The ROC Curve of the SVM model (The AUC is 0.721).

The 189 (22 skip metastasis and 167 non-skip metastasis) external independent samples were used to validate panel performance. The panel also achieved high accuracy in the validation set, with 91.5% accuracy (173/189 samples correctly classified, **Table 4**). Moreover, the specificity and NPV maintained a highly level, 93.1% and 97.6, respectively, in the validation set. On the contrary, the sensitivity and PPV were slightly decreased (71.4% and 45.5%, respectively). The AUC (Area under the curve) of SVM model in ROC Curve is 0.721 (**Figure 3**).

## Discussion

To date, PTC in 30%–80% of patients was accompanied by other forms of LNM (6). PTC with LNM was linked to local recurrence and cancer-specific mortality in certain patients (5, 25). The complications from re-operation of PTC, for example, hemorrhage, hypothyroidism, laryngeal nerve injury, and lymphatic leakage, were increased compared with the initial operation. Therefore, predicting LNM is important for surgeons to precisely determine neck dissections.

The underestimation of skip metastasis of PTC will result in incomplete lymph node dissection during the operation and ultimately lead to a poor prognosis in patients with PTC. However, when the CLN is negative, no further LLND will be performed unless lateral cervical lymphadenopathy is proven by preoperative biopsy and imaging modalities (26). However, a high false negative rate of LLNM by preoperative examination is found (27, 28). For example, about 30% of metastatic lymph nodes in level 3 could not be detected by preoperative examination in previous studies (27). Therefore, accurate assessment of the characteristics and mechanisms of the occurrence of skip metastasis to provide clinical guidance, such as close follow-up or more aggressive surgery, will improve the prognosis of thyroid cancer. In this research, the incidence and detailed characteristics associated with skip metastasis were evaluated by our group.

The reported rate of skip metastasis varies considerably, ranging from 5% to 25% (14). The difference in the frequency of skip metastasis might be due to the different regions and sample sizes. Given the small sample data in most previous studies, the accuracy of data is limited. In our study, we found that the frequency of skip metastasis was 13.3% (84/630), which was the largest sample analysis in skip metastasis. The high rate of skip metastasis indicates that we do not ignore the presence of skip metastasis in the clinical work, although the prognostic relationship between skip metastasis and PTC remains unknown. This form of metastatic spread to regional lymph nodes is also found in other cancer. The non-small-cell lung cancer and colorectal cancer were reported as a prognostic benefit (29, 30). On the contrary, the presence of metastases confers a poor prognosis when synchronous skip metastases are present in osteosarcoma (31). In previous studies, no significant difference in PTC-tumor-free survival was observed between the skip metastasis and non-skip metastasis groups (32). However, these results remain to be verified.

Machine learning (ML) is type of classifier learning from past date to predict future data. SVM is one of many ML methods (33). Compared to the other ML methods, SVM has very powerful ability to recognize subtle patterns in complex datasets (34). Moreover, Golub et al. had demonstrated the superior performance of SVM in classifying high-dimensional and low sample size data (35). Since 2000, the SVM has been used in many complex classification of cancer study, such as the cancer subtypes, the outcome prognosis, drug benefit prediction, tumorigenesis drivers, or a tumor-specific biological process (36–40). Therefore, in this study, the artificial intelligence of SVM was used to recognize many important factors in a variety of risk characteristics and establish a model to predict skip metastasis in PTC.

In this study, we found six independent predictors (tumor size ≤10 mm, located in the upper pole, Hashimoto’s thyroiditis, no BRAF^V600E^ mutation, extrathyroidal extension, and less number of CLND) for skip metastasis in PTC. Some of these clinicopathological or sonographic features were considered as risk factors for skip metastasis in previous studies, but the results were disputed. For example, the positive correlation between upper location and skip metastasis has been shown in many earlier studies (14, 32, 41). This connection may be interpreted by the anatomical structure, which could migrate along the superior thyroid artery to LLN and leap over CLN. Some studies have reported that the skip area in level II or/and III is a predictor of skip metastasis (41, 42). In addition, tumor size ≤ 10 mm, well-defined margin and

extrathyroidal extension were recognized as predictors of skip metastasis (28, 41–43). Furthermore, we observed that skip metastasis could easily occur in PTC patients with Hashimoto’s thyroiditis and without BRAF^V600E^ mutation. What’s more, the less number of CLND as a predictor was found in both this study and previous studies (44).

To date, the leading causes of skip metastasis are not completely clarified yet. Skip metastasis may be due to the following reasons: first, nodal metastasis bypasses, without normal anatomical lymphatic channels, were observed (14, 45). Thus, less aggressive nodules (tumor size <10 mm) are more easily characterized by skip metastasis as they passes through the metastasis bypasses before invading the central lymph node compartment. Previous reports have suggested that the absence of BRAF^V600E^ mutation and Hashimoto’s thyroiditis-positive PTC were associated with a less aggressive disease (43). Second, extrathyroidal extension or neck treatment alters the normal lymphatic channels and induces skip metastasis; In addition, inadequate CLND sampling may cause false negative findings. For example, metastatic lymph node missed during LND could cause a negative finding. Therefore, a less number of CLND is a risk factor of skip metastasis.

A predictive model for skip metastasis was first established in this research. This model has a good performance and high accuracy, indicating its strong clinical feasibility. In addition, the model has high specificity and NPV, which indicates that the model has a high ability to exclude patients with non-skip metastasis. If the model predicts a non-skip metastasis, then the probability that this result is correct is 98.9%. Therefore, patients with non-skip metastasis can be basically excluded when they are predicted negative by this model. Furthermore, although the sensitivity decreased in the validation set (71.4%) compared with the training set (86.4%). The predictive sensitivity of model was significantly higher than that of each single factor. However, the disadvantage of the model’s PPV (45.2%) is not obvious. Since there were few guidelines to support perform prophylactic LLND in the cN0 thyroid cancer with high risk of skip metastasis, this model focused on identifying the non-skip metastasis patients and ensure the higher specificity and NPV. Therefore, if the model predicts a high risk of skip metastasis, close follow-up or more aggressive surgery could be considered according to the status of disease.

The current study has several limitations. First, this study is a single-center retrospective study; hence, the results may have some differences from other studies. For further research, we are undertaking a multicenter prospective study. Second, although the model can be more convenient and accurate to guide the operation, whether the model has survival benefits remains unknown. Therefore, more follow-up studies will be conducted in the future.

In conclusion, we found that the rate of skip metastasis was 13.3% (84/631), which was the largest sample analysis so far. We screened six predictors of the skip lateral lymph node metastasis: tumor ≤10 mm, locating in the upper pole, Hashimoto’s thyroiditis, extrathyroidal extension, less number of CLND and without BRAF^V600E^ mutation. This study is the first to explore the association of Hashimoto’s thyroiditis and BRAF^V600E^ mutation with skip metastasis. Moreover, we established an effective predictive model for skip metastasis in PTC. This model can accurately distinguish the skip metastasis in PTC using a simple and affordable method, which may have the potential for daily clinical application in the future.

## Data Availability Statement

The raw data supporting the conclusions of this article will be made available by the authors, without undue reservation.

## Ethics Statement

The studies involving human participants were reviewed and approved by The Ethics Committee of the First Affiliated Hospital of Wenzhou Medical University. The patients/participants provided their written informed consent to participate in this study.

## Author Contributions

GZ and YC contributed to the conception and design of the research. BS and CJ contributed to collected the clinical information. SZ, QW and DZ contributed to performed the statistical analysis and make the charts. YC, ZZ and SX contributed to establish the model. All authors contributed to manuscript revision, and all authors read and approved the submitted version.

## Funding

This work was kindly supported by the grant from Major Science and Technology Projects of Zhejiang Province (NO.2015C03052); National Natural Science Foundation of China-Zhejiang Joint Fund (No. U20A20382); National Natural Science Foundation of China (No. 81703575, 82103199, 81827170 and 81802673); Key Research and Development Program of Zhejiang Province (No. 2021C03081).

## Conflict of Interest

The authors declare that the research was conducted in the absence of any commercial or financial relationships that could be construed as a potential conflict of interest.

## Publisher’s Note

All claims expressed in this article are solely those of the authors and do not necessarily represent those of their affiliated organizations, or those of the publisher, the editors and the reviewers. Any product that may be evaluated in this article, or claim that may be made by its manufacturer, is not guaranteed or endorsed by the publisher.

## Supplementary Material

Supplementary Table 1 The baseline data of training set and validation set.

## Abbreviations

PTC, papillary thyroid carcinoma; SVM, support vector machine; CLN, central lymph node; LLN, lateral lymph node; CLNM, central lymph node metastasis; LLNM, lateral lymph node metastasis; CLND, central lymph node dissection; LLND, lateral lymph node dissection; CI, confidence interval; OR, odds ratio; PPV, positive predictive value; NPV, negative predictive value.
