# Supplementary material for: A Novel and Effective Model to Predict Skip Metastasis in Papillary Thyroid Carcinoma Based on a Support Vector Machine
Source: Front Endocrinol (Lausanne). 2022 Jul 5;13:916121. doi: 10.3389/fendo.2022.916121 (PMC9295388; doi:10.3389/fendo.2022.916121)
Supplement: Supplementary Table 1 — The baseline data of training set and validation set. [file Table_1.docx]

Table S1. The baseline data of training set and validation set.

|  | Training Set | Validation Set | F/t | P value |
| --- | --- | --- | --- | --- |
|  | Patient (n=630) | Patient (n=189) |  |  |
| Age at diagnosis(years) | 45.28±12.48 | 46.69±12.11 | -1.379 | 0.168 |
| Tumor size(mm) | 14.25±8.88 | 12.25±7.74 | 2.795 | 0.005^*^ |
|  |  |  | 7.885 | 0.005^*^ |
| ≤10 (n, %) | 267 | 102 |  |  |
| ＞10 (n, %) | 363 | 87 |  |  |
| Sex |  |  | 2.125 | 0.16 |
| Male (n, %) | 204 | 72 |  |  |
| Female (n, %) | 426 | 117 |  |  |
| Skip metastasis |  |  | 0.37 | 0.622 |
| Yes (n, %) | 84 | 22 |  |  |
| No (n, %) | 546 | 167 |  |  |
| Hashimoto's thyroiditis |  |  | 0.028 | 0.922 |
| Yes (n, %) | 147 | 43 |  |  |
| No (n, %) | 483 | 146 |  |  |
| Tumor location |  |  | 5.029 | 0.028 |
| Upper (n, %) | 193 | 42 |  |  |
| Lower/middle/isthmus (n, %) | 437 | 147 |  |  |
| Extrathyroidal extension |  |  | 0.91 | 0.35 |
| Yes (n, %) | 172 | 45 |  |  |
| No (n, %) | 458 | 144 |  |  |
| BRAF mutation |  |  | 3.906 | 0.05^*^ |
| Mutation(n, %) | 473 | 155 |  |  |
| No mutation(n, %) | 157 | 34 |  |  |
| Margin |  |  | 0.475 | 0.491 |
| Well-defined (n, %) | 396 | 124 |  |  |
| without a well-defined margin (n, %) | 234 | 65 |  |  |
| Central lymph nodes dissected number | 7.57±5.71 | 6.95±5.18 | 1.344 | 0.179 |
